# Increased Cannabinoid Receptor 1-Immunopositive Perisomatic Input of Principal Cells in Human Epileptic Patients in Focal Cortical Dysplasia Type IIB

**DOI:** 10.3390/ijms27146326

**Published:** 2026-07-16

**Authors:** Cecília Szekeres-Paraczky, Péter Szocsics, Loránd Erőss, Orsolya S. Mihály, Zsófia Maglóczky

**Affiliations:** 1 Szentágothai János Doctoral School of Neuroscience, Semmelweis University, 1085 Budapest, Hungary; paraczky.cecilia@koki.hu (C.S.-P.);; 2 Human Brain Research Laboratory, Institute of Experimental Medicine, HUN-REN, 1083 Budapest, Hungary; 3 Department of Functional Neurosurgery and Center of Neuromodulation, National Institute of Mental Health, Neurology and Neurosurgery, 1145 Budapest, Hungary; 4 Department of Pathology, St. Borbála Hospital, 2800 Tatabánya, Hungary

**Keywords:** perisomatic inhibition, dysmorphic neurons, human cortex, cannabinoid receptor 1, focal cortical dysplasia, interneurons

## Abstract

Drug-resistant epilepsy is often associated with a neurodevelopmental disorder, focal cortical dysplasia (FCD). As demonstrated by numerous studies, defects in perisomatic inhibition may play a role in the formation of seizures. In our previous study, we observed an increase in parvalbumin-immunopositive perisomatic input. It has been observed that other perisomatic inhibitory cells expressing cannabinoid receptor type-1 (CB1R) and containing cholecystokinin (CCK) sprout in epileptic hippocampi. Therefore, we have investigated whether CB1R-immunopositive innervation has also changed in FCD. FCDIIB surgical samples were compared to controls with short post-mortem delay. The perisomatic input contacting principal cells was examined by NeuN-CB1R double immunostaining. Quantification in 3D was performed using a confocal fluorescence microscope. The results show that the CB1R-immunopositive synaptic input of principal cells is significantly larger in FCD cases. This condition may be an adaptive response to abnormal activity, or it may be a pre-existing pathology that is exacerbated by seizures. The reorganisation of the perisomatic inhibitory system has the potential to further increase the seizure probability in both cases. It should be noted that alterations in both PV- and CCK-CB1R-expressing basket cells may be implicated in seizure generation.

## 1. Introduction

### 1.1. Drug-Resistant Epilepsies

The global prevalence of drug-resistant epilepsy poses significant challenges due to its unpredictable outcomes and limited treatment options [1,2,3,4]. As demonstrated in the relevant literature, drug-resistant epilepsy cases that present with early age-appearing seizures frequently have a focal cortical dysplasia (FCD) background [5,6,7,8]. FCD has been identified as the most prevalent diagnosis among surgically treated epileptic children [8,9]. Furthermore, in 2018 statistical data from 16 European epilepsy centres revealed that drug-resistant subjects had a higher number of recent cases associated with dysplasia than with hippocampal sclerosis (HS) [10]. It is therefore vital that research into the pathomechanism of FCDs is given greater priority.

FCDs are neurodevelopmental disorders that can occur at different stages of neural development [11,12,13,14,15,16]. Seizures typically manifest during infancy and early childhood [5,6,7,8]. A taxonomic classification system has been proposed, which subdivides the condition into multiple subgroups, according to the various anatomical discrepancies that occur as a consequence of the developmental deficit [13,15,17,18,19,20].

Type I FCDs (types IA, IB, IC) are characterised by abnormal radial and/or tangential cortical lamination and minor cellular abnormalities, while type III FCDs are identified by irregular cortical lamination adjacent to various types of other lesions (types IIIA, IIIB, IIIC, IIID). The FCD type II group under investigation is characterised by severe local dyslamination and the presence of abnormal cell types. Cytomegalic interneurons, as well as dysmorphic neurons and abnormal glial cells, appear in both type IIA and IIB, while so-called balloon cells are exclusively specific to type IIB [13,15,17,18,19,20]. It has been demonstrated that FCD type II specific cell types express immature cell markers, thus indicating that the deviation from normal development commences during the proliferation phase, i.e., the early stage of neural maturation [11,21]. Genetic findings indicate that the mammalian target of rapamycin (mTOR) signalling pathway is significantly damaged in the FCD type II group [5,22,23,24]. This may offer a partial explanation for the observed anatomical and molecular characteristics. The atypical cell types of the FCD II group are distinguished by their abnormal location, orientation, and size, as well as their atypical dendritic processes [15,19,20,25].

### 1.2. Perisomatic Inhibition Concerning Epilepsy

The regulation of synchronous principal cell firing by basket cells can be subdivided into two principal aspects: the fast and firm, and the more delayed, fine-tuning regulation. In the aforementioned process, parvalbumin (PV)-immunopositive axo-axonic and basket cells are implicated. In the latter case, the involvement of cholecystokinin (CCK)- and cannabinoid receptor type 1 (CB1R)-immunopositive basket cells has been demonstrated. Studies posit that the healthy functioning of the cortical pyramidal cells is contingent on a normal balance between these two systems [26,27].

In our previous investigations, altered PV-immunopositive perisomatic input was found in human hippocampal (HC) and cortical samples of surgically treated patients with temporal lobe epilepsy (TLE) and FCDIIB [28,29,30,31,32]. In the context of HC samples preserved or sprouting perisomatic inhibitory input was identified [28,30]. These conditions can lead to increased synchronous firing of principal cells and consequently an enhanced probability of generating seizures [28,29,30,31,32].

### 1.3. Epilepsy Studies on CCK-CB1R-Immunopositive Perisomatic Inhibition

Although the synchronous function of principal cells is primarily influenced by PV-immunopositive perisomatic inputs, CCK-CB1R-immunopositive retrograde signalling may also play a significant role in the maintenance of normal cortical function [26,27,33,34,35,36,37].

In 2022, Lazarini-Lopes and Silva-Cardoso provided a synopsis of the experimental findings derived from the utilisation of diverse animal models, encompassing genetic, pharmacological, electrophysiological and febrile models. The findings from the various model types were not universally consistent; on some occasions, a decline in CB1R immunolabeling and protein/mRNA levels was observed, while in numerous other instances, an increase was detected [38]. The majority of research groups investigated the hippocampus [32]; however, the investigation of neocortical areas was largely overlooked.

A human study demonstrated a significant decrease in the ratio of CB1R-containing glutamatergic terminals in epileptic hippocampus tissue samples [35]. Another study examining FCD samples found a lower density of GABAergic neurons in the dysplastic areas [39]. In our previous CB1R study examining TLE, an increase in the number of GABAergic axons expressing CB1R in the sclerotic hippocampi, as well as elevated CB1R levels, was observed [36,40].

Consequently, the majority of studies have focused on alterations in CB1R in animal models of epilepsy [38]. A limited number of anatomical and electrophysiological studies have been conducted on human TLE samples [32,35,36], but human FCD has received relatively little attention in research to date [41,42].

### 1.4. Our Present Investigation

The focal point of our FCD studies pertained to the perisomatic input of layer 3 and 5 principal cells in FCDIIB patients. Dysmorphic neurons are most likely modified pyramidal cells [5,43,44] and most of them are immunopositive for NeuN and SMI32 too [5,9,25]. Consequently, we employed the term “giant” to denote principal cells that exhibited abnormally large cell bodies and aberrant dendritic arborizations in FCD samples. Although FCDIIB-specific balloon cells also exhibit similar characteristics, they typically have few thin processes and are predominantly located in layer 6 and the white matter [5,43,44]. Consequently, the probability of their inclusion in the measured cells is minimal.

Following the demonstration of a significant change in PV-positive perisomatic input in six FCDIIB cortical samples [29], the objective was to investigate whether CB1R-immunopositive perisomatic innervation had also changed in FCDIIB. Accordingly, an examination was conducted to determine the number of CB1R-immunopositive perisomatic terminals on the principal cells, comparing with short post-mortem control samples.

## 2. Results

### 2.1. Qualitative Description of Control and FCD Tissue Samples

#### 2.1.1. Control Tissue

Based on our earlier studies [30,31,36,45], well-preserved post-mortem control samples (*n* = 6) with short PMI were selected.

**Figure 1:**
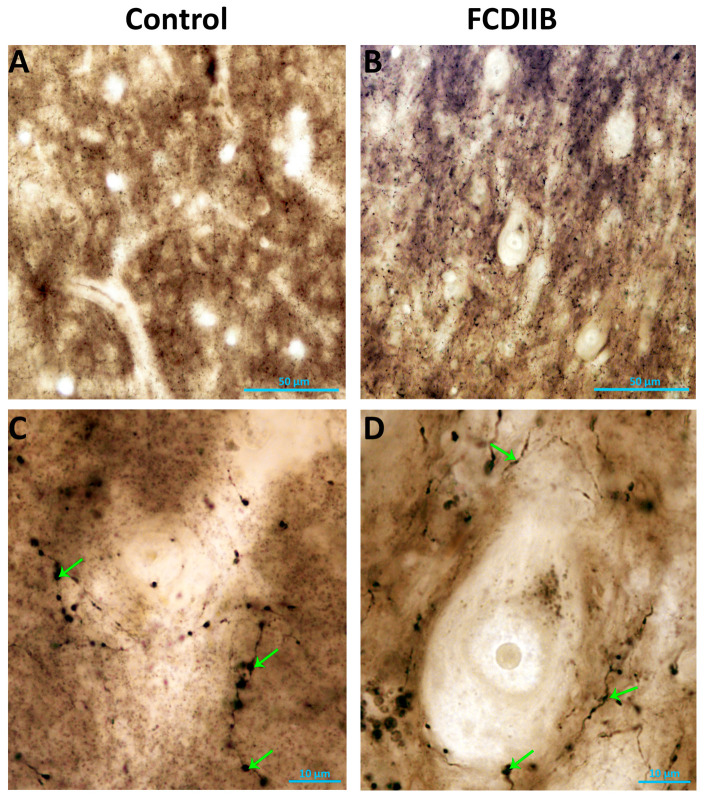
CB1R-immunopositive elements in a control sample (**A**,**C**) and in an epileptic patient with FCDIIB in frontal cortex (**B**,**D**). Green arrows show the CB1R-containing elements exhibiting a meshwork pattern surrounding the neuronal cell bodies. Scales: (**A**,**B**) 50 μm; (**C**,**D**) 10 μm.

**Figure 2:**
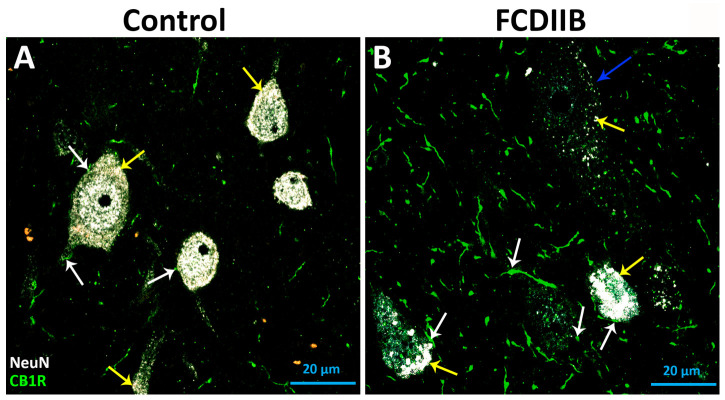
NeuN-CB1R double-immunostained cells in the prefrontal cortex of control (**A**) and FCDIIB (**B**) epileptic subjects in layer 3 with confocal fluorescence imaging. White arrows: CB1R-containing elements, forming presumable synapses; blue arrows: presumable dysmorphic neuron; yellow arrows: autofluorescent lipofuscin drops. It is the consequence of non-specific binding that the red blood cells appear in orange. Scales: 20 μm.

The perisomatic input was examined CB1R (Figure 1) and NeuN-CB1R double immunostaining (Figure 2). CB1R staining in control samples has revealed the presence of several terminals exhibiting a meshwork pattern surrounding the neuronal cell bodies.

**Figure 3:**
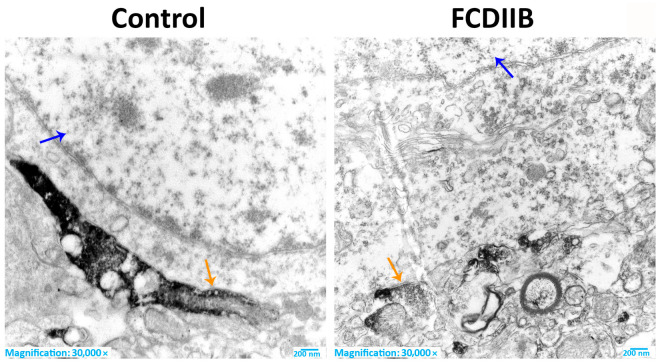
CB1R-immunostained sections of the somata of principal cells in prefrontal cortex of control and FCDIIB subjects in layer III by electron microscopy. The CB1R-immunopositive perisomatic terminals are establishing symmetrical synapses with the principal neurons (orange arrows). Blue arrows show the nuclei of the principal cells. Scale: 200 nm.

The perisomatic CB1R-immunopositive terminals were also examined using an electron microscope. The presence of symmetrical synapses on the cell bodies has been established (Figure 3).

**Figure 4:**
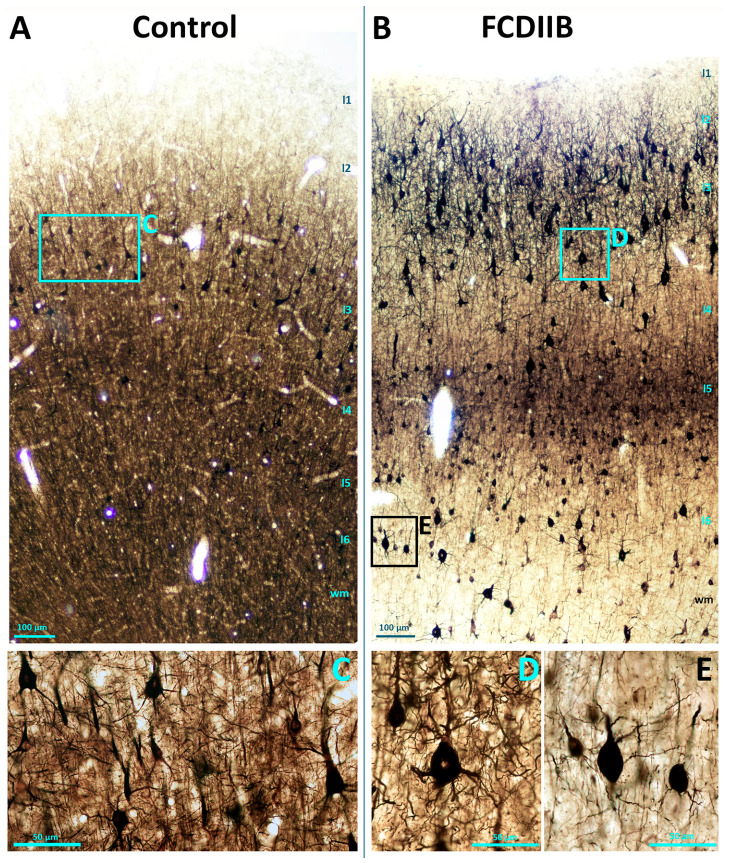
Non-phosphorylated neurofilament H (SMI32)-immunopositive elements in a control sample (**A**,**C**) and in an epileptic patient with FCDIIB in frontal cortex (**B**,**D**,**E**). SMI32-immunostaining labels principal cells with high specificity. Control principal cells from layer 3 are shown in the (**C**) panel. There are numerous giant (presumably dysmorphic) neurons throughout the FCD cortex (**B**), layer 3–4: panel (**D**), white matter: panel (**E**). These giant neurons have abnormally thick and numerous dendrites as well as thin, irregular processes (**D**,**E**). Scales: (**A**,**B**) 100 µm; (**C**–**E**) 50 µm.

The principal cells were examined by SMI32-immunostaining (Figure 3). The cortical lamination in the control cortices was found to be normal. The SMI32-labelled neurons exhibited normal orientation and morphology (Figure 4A,C).

#### 2.1.2. FCDIIB Samples

The tissue samples from FCD subjects exhibited some heterogeneity in terms of lamination and the prevalence of atypical cell types. In the majority of cases, the cortical lamination was found to be slightly abnormal, with local dyslamination present. For the purpose of quantitative analysis, regions exhibiting distinguishable cortical layers were selected for comparison with control tissues (Figure 4B).

The presence of dysmorphic neurons with abnormal morphology and enlarged cell bodies in the cortex is a common feature of FCD type II pathology [5,18,19,20]. In the present investigation, these neurons were found throughout the cortex but were predominantly located in layers 3 and 5–6 (Figure 4B,D,E). The subjects were found to be immunopositive for SMI32 (Figure 4).

The FCDIIB samples differed from each other: HE117, HE177, HE180 and HE239 exhibited a reduced number of giant neurons and balloon cells. Conversely, HE199 and HE220 exhibited numerous pathological giant cell types.

The NeuN antibody is a widely utilised tool in the field of neuroscience, as it is capable of detecting neurons in their nucleus and, to a limited extent, in their cytoplasm, but not in their entire processes. Consequently, this staining method is considered appropriate for general quantifications [46].

### 2.2. Quantitative Examination of CB1R-Immunopositive Perisomatic Terminals in the Control and FCDIIB Tissue Samples

Perisomatic inhibitory input means the terminals on the cell bodies and proximal dendrites originating from PV-and CCK-CB1R-expressing basket cells, or on the axon initial segments (AIS) by the axo-axonic cells [27,34]. In the present study, CB1R-immunopositive terminals located in close proximity to the cell body and up to 25 µm on the dendrites were enumerated and specified of the unit area (µm^2^) of the cell surface, as well as unit dendritic length (µm). Table 1 and Table 2 show the number of CB1R-immunopositive perisomatic terminals contacting the measured cell bodies (Table 1) and up to 25 µm on the dendrites per number of measured cells (Table 2).

#### 2.2.1. Comparison of Pooled Dendritic Data of Control and FCDIIB Subjects in Layer 3 and 5

**Figure 5:**
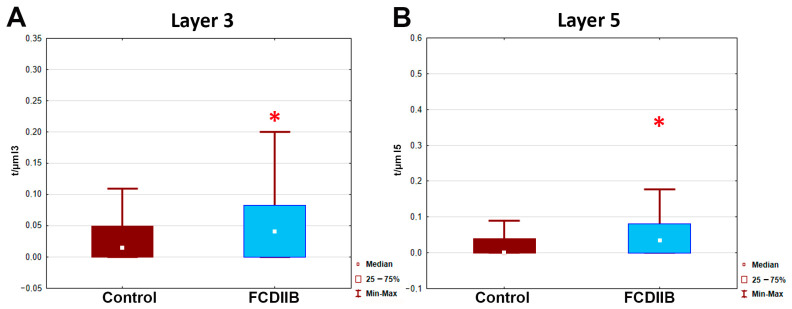
Boxplots show a statistical comparison of the number of CB1R-immunopositive terminals per unit dendritic length (µm) in control (claret box) and epileptic FCDIIB (blue box) subjects in layer 3 (**A**) and layer 5 (**B**) by Mann–Whitney U test. The values in both layers show significant differences between the two groups. Layer 3: Mann–Whitney U = 9075, N1 = 152, N2 = 161, *p* = 4.79 × 10^−5^; Layer 5: Mann–Whitney U = 9075, N1 = 136, N2 = 135, *p* = 1.9 × 10^−5^ (**A**,**B**). * *p* < 0.05. N1: the number of cells with measured proximal dendrites in control samples; N2: the number of cells with measured proximal dendrites in FCDIIB samples.

The data from the FCDIIB and control samples were then amalgamated according to layers 3 and 5. The Mann–Whitney U test was employed to compare the number of CB1R-immunopositive terminals per unit proximal dendritic length (µm) between control and epileptic samples. A comparison of pooled datasets revealed that the number of CB1R-immunopositive terminals per unit dendritic length (µm) is significantly higher in FCD cases. Layer 3: Mann–Whitney U = 9075, N1 = 152, N2 = 161, *p* = 4.79 × 10^−5^; Layer 5: Mann–Whitney U = 9075, N1 = 136, N2 = 135, *p* = 1.90 × 10^−5^ (Figure 5A,B). * *p* < 0.05 The increase is higher in layer 3 than in layer 5. N1: the number of cells with measured dendrites in control samples; N2: the number of cells with measured dendrites in FCDIIB samples (Figure 5).

#### 2.2.2. Comparison of Pooled Data of Control and FCDIIB Subjects per Layer Regarding of the Apical and Basal Dendrites

Data from FCDIIB and control samples were pooled per layer and the number of CB1R-immunopositive terminals per unit proximal dendritic length unit (µm) were compared using the Mann–Whitney U test selectively on apical and basal dendrites. The comparison of pooled data showed that the number of perisomatic CB1R-immunopositive terminals per dendrite length unit (µm) is significantly higher on the apical dendrites in FCD cases: Layer 3: Mann–Whitney U = 9146, N1 = 152, N2 = 161, *p* = 1.90 × 10^−5^; Layer 5: Mann–Whitney U = 6847, N1 = 136, N2 = 135, *p* = 3.40 × 10^−5^, but not on the basal dendrites.

#### 2.2.3. Comparison of Pooled Data of Control and FCDIIB Subjects per Layer

**Figure 6:**
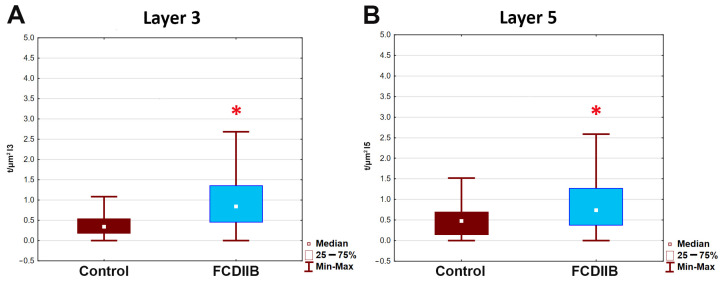
Boxplots show a statistical comparison of the number of CB1R-immunopositive perisomatic terminals per unit area (μm^2^) of soma surface in control (claret box) and FCDIIB (blue box) subjects in layer 3 (**A**) and layer 5 (**B**) by Mann–Whitney U test. Both layers show significant differences in the two measured populations. Layer 3: Mann–Whitney U = 5798.00, N1 = 152, N2 = 161, *p* = 8.64 × 10^−16^; Layer 5: Mann–Whitney U = 5842, N1 = 136, N2 = 135, *p* = 2.29 × 10^−7^ (**A**,**B**). * *p* < 0.05 The increase is higher in layer 3 than in layer 5. N1: the number of cells counted in control samples; N2: the number of cells counted in FCDIIB samples.

The data from the FCDIIB and control samples were combined into a single dataset for each layer. The number of CB1R-immunopositive perisomatic terminals per unit area (µm^2^) of cell surface were then compared using a Mann–Whitney U test. The comparison of pooled data demonstrated that the number of perisomatic CB1R-immunopositive terminals per unit area (µm^2^) of cell surface is significantly higher in FCD cases. Layer 3: Mann–Whitney U = 5798.00, N1 = 152, N2 = 161, *p* = 8.64 × 10^−16^; Layer 5: Mann–Whitney U = 5899.00, N1 = 137, N2 = 135, *p* = 2.44 × 10^−7^ (Figure 6A,B). The increase is higher in layer 3 than in layer 5. N1: the number of counted cells in control samples; N2: the number of counted cells in FCDIIB samples (Figure 6).

## 3. Discussion

### 3.1. Possible Role of CB1R-Expressing Innervation of Principal Cells in Epileptic Disfunction

It has been established that excessive synchronous firing of neuronal populations can result in abnormal brain activity and epileptic seizures [47,48]. This pathological action of principal neurons has been demonstrated to be closely related to the dysfunction of the perisomatic inhibitory system [40,49,50,51,52,53].

The dysfunction of CCK/CB1R-positive basket cells has been linked to mood disorders [26,27], yet research in the human FCD cortex remains limited. However, given the neuroprotective function of the endocannabinoid system [35,54,55], it cannot be discounted that the reorganisation of CCK/CB1R-immunoreactive elements may be a contributing factor in epileptic conditions [32,35,36,40]. Furthermore, similarly to the PV-immunopositive elements, an increased number of CB1R-immunopositive perisomatic terminals may also contribute to the enhanced synchronous activity. The literature suggests that in the basolateral amygdala, both PV- and CCK/CB1R-immunopositive basket cells can prevent or delay the firing of principal cells with a high degree of efficiency [34,56].

### 3.2. Evaluation of the Qualitative Results

The control (with short PMI) and surgical FCDIIB tissue samples were processed in an identical manner post-removal from the brain. As demonstrated by earlier studies and recent qualitative analyses, the two types of brain tissue are appropriate for their appropriate comparisons [29,30,31,45,57,58,59,60,61]. The six region-matched control samples exhibited homogenous anatomical features with regard to neuronal cell morphology. However, the FCDIIB tissues from subjects diagnosed with epilepsy exhibited pathological abnormalities with respect to cell morphology and lamination properties. Immunohistochemical and structural preservation was appropriate and similar in both examined groups.

The FCDIIB tissue samples exhibited significant morphologic heterogeneity, characterised by the presence of varying proportions of abnormal cell types. The potential causes of these observations (e.g., the duration of epilepsy, medical treatments, and the location of the sampling area) were discussed in our previous paper according to the literature [29,62,63].

#### The Significance of Subject Age

The surgical and post-mortem tissue samples available for examinations are not suitable for matching by age. However, the results of previous studies have indicated that CB1R expression in control samples with a short post-mortem interval does not vary significantly with age [35,64,65]. Epilepsy disease exerts a considerably more substantial influence on the configuration of the perisomatic inhibitory system [30]. These alterations diverged from those observed in the control samples in all instances, demonstrated stability, and were not arbitrary [66]. It has been confirmed in several studies that CB1R is remarkably stable and, provided that structural integrity is maintained in the tissue, does not degrade within 4–5 h post-mortem [35,65]. Furthermore, in all cases, subjects were included in the study only if adequate preservation was observed upon comparison of the initial light-microscopic immunostainings. Consequently, tissues that were imperfectly fixed due to sclerosis of the cerebral blood vessels are not utilised.

Numerous studies have previously investigated age-related alterations in the quality and quantity of CB1R. The results are highly contradictory in terms of both the objects of study—the quantity, distribution and function of receptors—and the methods and results [64,65,67,68,69,70]. Several articles analysed the hippocampus in detail across various diseases. Most articles supported the finding that the relationship between CB1-R levels and age varies across different diseases and brain regions [64,67,70]. Some studies revealed that the number of receptors in several brain regions decreases somewhat—not consistently significant across all cases—with advancing age [65,67,70]. It is possible that a similar decrease may also occur in the samples, however, this trend is not clearly observable in the control subjects examined in this study. (see Table 1). Furthermore, the standard deviation observed among the control subjects was found to be lower in comparison to the FCD samples with regard to the number of terminals counted (see Table 1). Furthermore, a marked increase in the number of receptors in surgical samples from patients with FCD was observed in both cortical layers that were examined. Since similar studies generally compared different brain regions and pathological conditions and performed in animal models, the data are not entirely comparable [35,36,38,39,41,42]. Nevertheless, the literature also emphasises that brain region and disease, rather than age, are the decisive factors influencing the quantity and distribution of CB1-R [64,67,69,70]. Therefore, we believe that our findings do indeed indicate a pathological increase in CB1-R number in FCD epilepsy.

### 3.3. Evaluation of the Quantitative Results

As indicated by the data pertaining to the detected dendrites and cell bodies, a considerable number of outliers were identified in the control samples, a finding that was analogous to the values observed in the FCDIIB samples. The diversity of outcomes is presumably attributable to the utilisation of Z-stack sampling, given the inclusion of dendrites of varying numbers and dimensions in the recordings. A further disadvantage of this 3D counting technique is that the giant cells were not included in the statistics because their dimensions exceeded the capacity of the sampling area. However, our qualitative microscopic observations of FCDIIB samples indicate a heterogeneous distribution of terminals around the cells, independent of their size. In contrast, in our preceding study, a reduced number of PV-positive perisomatic processes were observed around normal-sized cells in comparison to giant cells or cells in the control group [29]. When the entire cell surface is taken into consideration, it is evident that the FCDIIB samples exhibit a greater degree of variation in comparison to the controls. This outcome aligns with our expectations, as it indicates that our controls were selected carefully.

The number of CB1R-positive perisomatic terminals was also compared in terms of proximal apical and basal dendrites separately. In the case of FCD samples, a greater number of CB1R-immunopositive terminals were found to be associated with the proximal basal and apical dendrites in both examined layers. The apical dendrites received significantly more CB1R-positive input than the basal dendrites (Table 2). The perisomatic inputs on the apical dendrites of principal cells have been demonstrated to play a significant role in regulating population synchrony activity [26,27]. Therefore, this phenomenon may be the result of compensation for pathological excitatory activity.

The release of excitatory or inhibitory neurotransmitters is reduced in a CB1-dependent manner, suggesting that the endocannabinoid system may play a significant role in modulating nervous system processes associated with increased activity. In addition to structural reorganisation of innervation, alterations in cannabinoid receptor expression may also occur as a consequence of seizures, which could compromise the neuroprotective and anticonvulsant endocannabinoid signalling pathway [35]. Although PV-immunopositive input plays a major role in regulating synchronous firing [27], increased CB1R-immunopositive perisomatic inhibitory input may also contribute to triggering further seizures by supporting synchronous activity.

A general increase in the termination pattern has been observed in the CB1R-immunopositive terminals when the dendritic and cellular surfaces were examined by sampled cortical regions. However, given the limited sample size, it is not possible to draw definitive conclusions about the differences in brain regions based on these results.

### 3.4. Limitations

Despite the fact that short post-mortem intervals can be considered appropriate for comparisons with surgical tissue [30,31,57,58,59,61], it was not possible to eliminate the possibility that the differing ages of the control subjects and perimortem neuronal network changes could modify CCK/CB1R-containing terminals [71]. However, research into epilepsy has indicated that the amount of CB1R-immunopositive inhibitory elements increases, or remains unchanged after death [35,40].

Although the fact that we worked with a sample number that is suitable to the basic statistical requirements, if possible, we need to expand our studies with tissue samples of additional FCDIIB subjects. The existing literature offers a paucity of knowledge regarding the alterations of CB1R-immunopositive perisomatic input in subjects affected by FCD and in animal models. However, several results are consistent with our findings in animal models [38,40,72] as well as in human subjects [35,36,40].

The number of synapses formed by the examined perisomatic CB1R-expressing terminals with the cell body and proximal dendrites remains to be determined. The validation of these observations will require quantitative electron microscopy studies to be conducted in future. However, close-contact terminals around the cell body most likely form functional synapses [73,74,75]. Furthermore, an increased prevalence of inhibitory synapses relative to excitatory synapses has been observed in the perisomatic region [27,31,75].

## 4. Materials and Methods

### 4.1. Obtaining the Human Tissue

#### 4.1.1. FCDIIB Patients

Patients (*n* = 6) with drug-resistant epilepsy accompanied by focal cortical dysplasia (FCDIIB) as defined by the International League Against Epilepsy classification [13,17] underwent surgical intervention at the National Institute of Mental Health, Neurology and Neurosurgery in Budapest, Hungary, as part of the Hungarian Epilepsy Surgery Program. Prior to undergoing surgery, written informed consent for participation in the study was obtained from each patient. The seizure focus was identified by multimodal studies, including video-EEG monitoring, magnetic resonance imaging, single photon emission computer tomography, and/or positron emission tomography. The samples of epileptic tissue that were identified, and which correspond to the frontal (BA46 equivalent), parietal (BA7 equivalent), and occipital (BA18 equivalent) cortical areas, were surgically removed. Following surgical removal, the epileptic tissue was immediately divided into 0.5–0.6 cm thick blocks and immersed in a Zamboni fixative solution comprising 4% paraformaldehyde, 0.05% glutaraldehyde, and 0.2% picric acid in 0.1 M PB (the same fixative solution used for the control brain perfusion). The fixative was substituted at hourly intervals for a fresh solution, with constant agitation applied throughout a 4 h period at room temperature. Thereafter, the blocks were subjected to post-fixation overnight at 4 °C, utilising the same fixative devoid of glutaraldehyde [45]. For quantitative analyses, a sample of six FCDIIB patients was selected for further investigation (Table 3).

#### 4.1.2. Control Subjects

The study was approved by the ethics committee at the Regional and Institutional Committee of Science and Research Ethics of Scientific Council of Health (ETT TUKEB 15032/2019/EKU further modified in 2023) and performed in accordance with the Declaration of Helsinki. The five subjects who were under the control group have all passed away from causes unrelated to any brain disease. Furthermore, the clinical data and the autopsy results did not show any signs of neurological disorders. The removal of the control brains occurred between two and five hours following death. The cannulation of the internal carotid and vertebral arteries was then undertaken, after which the brains were perfused with physiological saline (1.5 L over 30 min) containing 5 mL of heparin. This was followed by the application of a Zamboni fixative (room temperature) solution comprising 4% paraformaldehyde and 0.2% picric acid in phosphate buffer (PB, pH 7.4) (4–5 L over 1.5–2 h). Subsequent to perfusion, 0.5–0.6 cm thick blocks were extracted from the cortical regions of the brain. The identification of regions was conducted in accordance with the Brodmann division 41 classification system, thereby encompassing Brodmann areas 7, 18, and 46. Subsequent to this identification, the regions were post-fixed in the Zamboni solution without the use of glutaraldehyde for a duration overnight at 4 °C [45]. For quantitative analyses, six cortical samples of five control subjects were selected (Table 4).

Thereafter, the samples were washed in 0.1 M PB and maintained in a 30% sucrose solution for a period of two days at 4 °C. Following this, they were frozen over liquid nitrogen and stored at −80 °C. Subsequently, 60 µm thick parallel sections were prepared from the blocks with a Leica VTS-1000 Vibratome for immunohistochemistry (Leica Biosystems Nußloch GmbH, Nußloch, Germany). The 60 µm-thick sections were meticulously extracted from the designated blocks and subsequently washed in PB four times. Thereafter, they were immersed in 30% sucrose for a period of 1–2 days at 4 °C, followed by three cycles of freeze–thawing over liquid nitrogen. The three-cycle freeze-thaw procedure over liquid nitrogen is utilised to enhance tissue permeability, thereby facilitating enhanced penetration of immunolabeling.

#### 4.1.3. The Effect of Fixation, Age, and PMI on Immunostaining

As demonstrated in earlier studies, the structural preservation of the tissue samples within 2–5 PMI is comparable with surgical samples (i.e., 0 h post-mortem delay) [30,31,36,45,59]. These results are consistent with the recent electrophysiological findings, which demonstrated that up to three hours after death, brain tissue remains suitable for electrophysiological recordings [58,76,77].

### 4.2. Immunohistochemistry

The sections were then subjected to an immunostaining procedure, which entailed the following steps. Following a thorough wash in 0.1 M PB, endogenous peroxidase activity was blocked by 1% H_2_O_2_ in Tris-buffered saline (TBS, pH 7.4) for a period of 10 min. The TBS was utilised for all washes (3 × 10 min between each antiserum) and for the dilution of the antisera. Non-specific immunostaining was blocked by 2% bovine serum albumin. We used antibodies against neuronal nuclei (NeuN, 1:2000 Chemicon raised in mouse, 1:4000 Sysy raised in guinea pig) and non-phosphorylated neurofilament H (SMI32, 1:4000, Biolegend raised in mouse) to detect the principal cells. All primary and secondary antibodies were tested by the producers for specificity. The concentration of the antibody was optimised to ensure sufficient penetration while minimising the labelling of excitatory terminals. Different CB1R antibodies were tested. Using an electron microscope, we found that a concentration of 1:500 is suitable for labelling inhibitory terminals but only labels a small number of excitatory terminals. However, epilepsy could alter mitochondrial health [78] and possibly produce false-positive results for CB1R-immunopositive terminals in confocal microscopy. Electron microscopy did not show any immunolabelled mitochondria (see Figure 3).

The sections were subjected to incubation for a period of either 24 h at room temperature or 48 h at 4 °C. For the purpose of light and electron microscopic visualisation of immunopositive elements, biotinylated anti-rabbit/mouse IgG (1:250, Vector) was employed as a secondary serum, followed by avidin-biotinylated horseradish peroxidase complex (1:250, Vector). Sections were subjected to incubation with 3,3′-diaminobenzidine tetrahydrochloride (DAB, Sigma, St. Louis, MO, USA) as a chromogen, dissolved in TRIS buffer. The immunoperoxidase reaction was then developed using 0.01% H_2_O_2_. Subsequently, the sections were subjected to a treatment with 0.5% osmium tetroxide in PB for a duration of 10 min. The dehydration process involved a series of steps, beginning with a 50% to absolute ethanol series, followed by an additional step of uranyl acetate at the 70% ethanol stage for a duration of 30 min. The samples were then mounted in Durcupan (ACM, Fluka, St. Louis, MO, USA). Conventional optical and electron microscopy techniques were utilised for the analysis of the samples.

In order to conduct the confocal microscopic investigations, the same methodology was employed in the preparation of sections as previously outlined, up until the point of the incubation of primary antibodies. The process of incubation with both primary antibodies was conducted concurrently (anti-NeuN raised in guinea pig at a dilution of 1:4000, anti-CB1R raised in rabbit at a dilution of 1:500). In order to facilitate the penetration of primary antibodies, 0.1% Triton was incorporated into the blocking solution. Following the incubation period, secondary antibodies with fluorophores were applied (Alexa488 anti-rabbit, 1:500, Invitrogen; Alexa 647 anti-guinea pig, 1:500, Jackson, both antisera raised in donkey) for a period of 3 h. To reduce autofluorescence, the samples were incubated with a solution of 3 mmol/L copper sulfate and 50 mmol/L ammonium acetate (pH 5) for a period of 40 min [79]. Then, we mounted them in Vectashield antifade medium (Vector, Newark, CA, USA).

### 4.3. Electron Microscopy

CB1R-DAB immunolabelled sections were prepared for qualitative electron microscopic analysis. Areas of interest in layer 3 were re-embedded and 60 nm ultrathin slices were prepared with an ultramicrotome (Leica EM UC6) and collected onto Formvar-coated single-slot copper grids, then counterstained with lead citrate (Ultrostain, Leica, Wetzlar, Germany). The samples were investigated using a transmission electron microscope (HT7800, Hitachi High-Tech Corporation, Hitachinaka, Japan) and the perisomatic synapses labelled by the chromogens were identified.

### 4.4. Quantitative Analysis

Sections were prepared and examined in the confocal microscope using double fluorescent NeuN-CB1R immunostaining. This was done in order to count the perisomatic CB1R-positive terminals around layer 3 and 5 NeuN-immunostained principal cells. Confocal microphotographs and z-stacks were obtained using a Nikon C2 Confocal Microscope equipped with 4× air (Plan Fluor NA = 0.13, WD = 17.2 mm, FOV = 3215.36 µm) and 60× oil (Plan Apo VC NA = 1.45, WD = 0.13 mm, FOV = 215.04 µm) objectives (Nikon Instruments Europe B.V., Amsterdam, The Netherlands). As previously stated, in order to ensure the validity of the statistical analysis, quantitative measurements were conducted in regions of the samples that exhibited distinguishable layers. The regions of interest (ROIs) encompassed the entire width of the cortical layers 3 and 5, with horizontal extents ranging from 250 to 350 µm. Z-stacks were constructed from a randomly selected array of NeuN-labelled cell bodies, with each cell body exhibiting clearly identifiable apical dendrites (averaging 20 cells per layer per sample). Depending on the size of the cells, the Z-stacks were 20–50 µm thick and had x-y dimensions of 118 × 118 µm. These stacks were then positioned within the designated regions of interest (ROI). The cell bodies were outlined at steps of 0.9 μm, which included the connecting dendrites that were visible in the image. Subsequently, the lengths of the dendrite segments were measured. The number of terminals counted on cell bodies or different dendrite types (apical, basal) was recorded. The surface area was calculated based on the available data. The CB1R-immunopositive perisomatic terminals were enumerated on cell bodies and up to 25 μm on dendrites in every third Z-plane. The number of CB1R-immunopositive perisomatic terminals that contacted the cell bodies of NeuN-labelled cells was counted in the ROI areas. Contact was determined if close contact was observed, and no hiatus was visible between the terminal and the cell body. CB1R-NeuN double-positive cells were excluded from the quantitative analyses. The number of CB1R-immunopositive inputs was specified to the unit area (µm^2^) of the cell surface and unit dendritic length (µm). The data pertaining to the measured cells in layers 3 and 5 were maintained in isolation. The ImageJ2-win64 program was utilised for the purpose of acquiring the requisite measurements [80]. The calculations and diagrams were created using Microsoft Excel 2016 and Statistica 13.4. In order to facilitate a meaningful comparison of the control and FCD cortices, non-parametric Mann–Whitney U tests were employed, with a significance level of *p* < 0.05.

## 5. Conclusions

In summary, it was found that the CB1R-immunopositive perisomatic input of principal cells in FCDIIB samples is significantly increased in comparison with control samples. Moreover, upon examination of the proximal segments of the dendrites, a more pronounced increase in the number of inhibitory terminals was observed on the apical dendrites in comparison to the basal dendrites. This may be indicative of a compensatory process that functions to counteract the pathological activity.

The present findings corroborate earlier results, which demonstrated that the alteration of perisomatic inhibition is more pronounced in layer 3 [29]. In contrast, Tóth et al. (2021) found that CB1R-positive perisomatic inhibitory inputs remained unchanged in focal cortical epilepsy [41]. However, the study’s authors used “lesion-free” tissues from patients diagnosed with brain tumours without epilepsy as control samples. Furthermore, the temporal cortex was examined by comparing various pathological samples [41].

In earlier studies, the presence of preserved CB1R-positive cell bodies and inhibitory terminals was identified in surgical TLE samples and control tissue with short PMI as well [35,36]. In accordance with preceding research, CB1R-immunopositive elements have been observed to manifest and reorganise in response to recurrent seizures [36,40]. The increased presence of CB1R-immunopositive terminals has been shown to result in chronic and excessive inhibitory processes [40]. Concomitant with the increased PV-immunopositive inhibition, this phenomenon may contribute to the intensification of the aberrant activity and the formation of epileptic seizures. The increased number of CB1R-immunoreactive terminals may also be indicative of changes in the endocannabinoid-mediated effects.

The extant data support our earlier results, suggesting that altered perisomatic input may be a key player in epileptic seizure generation. Collectively, these observations underscore the necessity for further investigation into the CB1R-immunopositive elements within the human FCD cortex, with a view to elucidating their potential roles in the pathophysiology of FCD.

## Figures and Tables

**Table 1:** Number of perisomatic terminals.

Layer 3				
	Control Case	Control (/Number of Cells)	FCD Case	FCD (/Number of Cells)
Number of perisomatic terminals/subjects	SKO3L BA46	136/24	HE117 parietal cortex/BA7	157/40
	SKO11L BA46	138/26	HE177 occipital cortex/BA18	217/23
	SKO13R BA46	96/31	HE180 frontal cortex/BA46	263/25
	SKO18R BA7	76/26	HE199 frontal cortex/BA46	161/22
	SKO18R BA18	31/25	HE220 frontal cortex/BA46	131/23
	SKO20L BA46	83/20	HE239 frontal cortex/BA46	187/28
Layer 3 mean number of terminals/mean number of cells in all subjects	-	93.33/25.33 SD: 40.29/3.56	-	186/26.83 SD: 47.71/6.79
Layer 3 mean number of terminals/1 cell	-	3.68	-	6.93
Layer 3 total number of terminals/total number of cells in all subjects	-	560/152	-	1116/161
**Layer 5**				
Number of perisomatic terminals/subjects	SKO3L BA46	67/13	HE117 parietal cortex/BA7	121/29
	SKO11L BA46	121/25	HE177 occipital cortex/BA18	165/20
	SKO13R BA46	88/26	HE180 frontal cortex/BA46	174/25
	SKO18R BA7	100/26	HE199 frontal cortex/BA46	199/22
	SKO18R BA18	39/25	HE220 frontal cortex/BA46	80/18
	SKO20L BA46	86/22	HE239 frontal cortex/BA46	96/21
Layer 5 mean number of terminals/total number of cells in all subjects	-	83.5/23.5 SD: 28.13/5.04	-	139.1/22.5 SD: 47.23/3.94
Layer 5 mean number of terminals/1cell	-	3.55	-	6.18
Layer 5 total number of terminals/total number of cells in all subjects	-	501/137	-	835/135
**Layers 3 and 5**				
Total number of terminals and cells	-	1061/289	-	1951/296

The table shows the number of CB1R-immunopositive perisomatic terminals contacting the measured cell bodies and proximal dendrites per number of measured cells in layers 3 and 5, respectively. It also shows the total numbers of counted terminals/cells of layers 3 and 5. SKO: control, HE: epileptic, L: Left, R: Right, BA: Brodmann Area, FCD: focal cortical dysplasia.

**Table 2:** Number of perisomatic terminals on proximal dendrites.

Criteria	Control (/All Apical Dendrites)	Control (/All Basal Dendrites)	Control (/All Dendrites)	FCD (/All Apical Dendrites)	FCD (/All Basal Dendrites)	FCD (/All Dendrites)
**Layer 3**						
Mean number of terminals/mean number of cells in all subjects	13.33/25.33 SD: 0.30	14.50/25.33 SD: 0.22	27.83/25.33 SD: 0.26	27.66/26.83 SD: 0.42	20/26.83 SD: 0.37	47.66/26.83 SD: 0.46
Mean number of terminals/1 cell	0.53	0.57	1.01	1.03	0.75	1.78
Total number of terminals/total number of cells in all subjects	80/152	87/152	167/152	166/161	120/161	286/161
**Layer 5**						
Mean number of terminals/mean number of cells in all subjects	9.66/23.5 SD: 0.40	10.66/23.5 SD: 0.44	20.33/23.5 SD: 0.42	21.66/22.5 SD: 0.41	13.5/22.5 SD: 0.29	35.17/22.5 SD: 0.37
Mean number of terminals/1 cell	0.42	0.47	0.89	0.96	0.6	1.56
Total number of terminals/total number of cells in all subjects	58/137	64/137	122/137	130/135	81/135	211/135
**Layers 3 and 5**						
Total number of terminals and cells	138/289	151/289	289/289	296/296	201/296	497/296

The table shows the number of CB1R-immunopositive perisomatic terminals contacting the proximal dendrites of the measured cells per number of measured cells in layers 3 and 5, respectively. It also shows the total numbers of counted terminals/cells of layers 3 and 5. FCD: focal cortical dysplasia.

**Table 3:** Data of the FCD patients involved in the quantitative analyses.

FCD Case	Age (Years)—At the Time of Surgery	Duration of Epilepsy (Years)	Gender	Pathological Classification	Removed Brain Area
HE117	26	16	male	FCDIIB	R parietal cortex/BA7
HE177	34.5	33	female	FCDIIB	R occipital cortex/BA18
HE180	17	15	female	FCDIIB	R frontal cortex/BA46
HE199	35	33.5	female	FCDIIB	R frontal cortex/BA46
HE220	48	39	male	FCDIIB	L frontal cortex/BA46
HE239	42	39.5	male	FCDIIB	L frontal cortex/BA46
Mean of parameters	33.75 SD: 11.08	29.33 SD: 11.05	-	-	-

The table shows the data of the FCD (HE) subjects. Areas from surgically removed samples of epileptic FCD patients are also indicated according to Brodmann. L: Left, R: Right, BA: Brodmann Area, FCD: focal cortical dysplasia, “HE”: the code name for patients diagnosed with FCD.

**Table 4:** Data of the control patients involved in the quantitative analyses.

Control Case	Age (Years)	Gender	PMI (Hours and Minutes)	Sampling Area
SKO3	59	female	5:05	L BA46
SKO11	77	male	2:55	L BA46
SKO13	60	female	3:25	R BA46
SKO18	85	male	2:52	R BA7, R BA18
SKO20	27	male	3:45	L BA46
Mean of parameters	61.60 SD: 22.31	-	3:36	-

The table shows the data of the control (SKO) subjects. Areas sampled during autopsy from control are indicated according to Brodmann. L: Left, R: Right, BA: Brodmann Area, PMI: post-mortem interval, “SKO”: the code name for post-mortem control subjects.

## Data Availability

All data and source code are available upon reasonable request from the corresponding authors. Some data are not publicly available due to ethical restrictions.

## Abbreviations

The following abbreviations are used in this manuscript:

AIS	Axon initial segment
BA	Brodmann Area
CB1R	Cannabinoid receptor type 1
CCK	Cholecystokinin
DAB	3,3′-Diaminobenzidine tetrahydrochloride
EEG	Electroencephalography
FCD/FCDIIB	Focal Cortical Dysplasia Type IIB
GABA	Gamma-aminobutyric acid
HC	Hippocampus
HE	Human epilepsy subject
HS	Hippocampal sclerosis
ILAE	International League Against Epilepsy
mRNA	Messenger ribonucleic acid
NeuN	Neuronal nuclei
PB	Phosphate buffer
PMI	Post-mortem interval
PV	Parvalbumin
ROI	Region of interest
SKO	Control subject
SMI32	Non-phosphorylated neurofilament H
TBS	Tris-buffered saline
TLE	Temporal lobe epilepsy
